# People making deontological judgments in the Trapdoor dilemma are perceived to be more prosocial in economic games than they actually are

**DOI:** 10.1371/journal.pone.0205066

**Published:** 2018-10-11

**Authors:** Valerio Capraro, Jonathan Sippel, Bonan Zhao, Levin Hornischer, Morgan Savary, Zoi Terzopoulou, Pierre Faucher, Simone F. Griffioen

**Affiliations:** 1 Department of Economics, Middlesex University, London, United Kingdom; 2 Institute for Logic, Language, and Computation, University of Amsterdam, Amsterdam, The Netherlands; 3 School of Engineering, Ècole Centrale Marseille, Marseille, France; Universidad Loyola Andalucia, SPAIN

## Abstract

Why do people make deontological decisions, although they often lead to overall unfavorable outcomes? One account is receiving considerable attention: deontological judgments may signal commitment to prosociality and thus may increase people’s chances of being selected as social partners–which carries obvious long-term benefits. Here we test this framework by experimentally exploring whether people making deontological judgments are expected to be more prosocial than those making consequentialist judgments *and* whether they are actually so. In line with previous studies, we identified deontological choices using the Trapdoor dilemma. Using economic games, we take two measures of general prosociality towards strangers: trustworthiness and altruism. Our results procure converging evidence for a perception gap according to which Trapdoor-deontologists are believed to be more trustworthy and more altruistic towards strangers than Trapdoor-consequentialists, but actually they are not so. These results show that deontological judgments are not universal, reliable signals of prosociality.

## Introduction

Human beings are constantly confronted with choices that have explicit moral dimensions. There has been much philosophical research about how to properly guide and judge these choices from an ethical point of view, leading to two popular traditions: *consequentialism* and *deontological ethics*. Consequentialism states that choices are to be assessed solely by their expected consequences and the states of affairs they bring about. Positions of this kind are often seen to have their intellectual predecessor in Utilitarianism as promoted by Jeremy Bentham and the work of John Stuart Mill, exemplified in Mill’s statement that “actions are right in proportion as they tend to promote happiness, wrong as they tend to produce the reverse of happiness” [1]. Along the lines of the consequentialist tradition, the consequences of an action (and thereby their moral value) have often been classified in terms of happiness or use–thus generally stating that an action is morally *good* if it brings about favorable consequences. Consequentialist views are often contrasted with deontological ethics. Here, the moral value of actions and choices is not to be evaluated solely on grounds of their consequences. An action is to be evaluated as morally good or bad if it is instantiating or violating certain ethical norms, respectively. Deontological ethics often claim to be of Kantian origin as Immanuel Kant prominently advocated an ethical framework relying on categorical norms and duties [2]. One example of fundamental importance is his *practical imperative* that demands respect for human beings as such: “So act that you use humanity, whether in your own person or in the person of any other, always at the same time as an end, never merely as a means” [2]. In Kantian and much of deontological ethics in general, it is thus prohibited to use human beings solely to achieve one’s goals–thereby demanding a certain respect for human life as such. Norms and duties of this kind have to be followed even if by that unfavorable overall consequences are to be expected. This philosophical discourse thus offers a possible background against which one can evaluate moral decision making and ethical behavior.

While the co-existence of the deontological and the consequentialist positions is defendable from a philosophical viewpoint, the persistence of deontological choices is puzzling from an evolutionary perspective, as deontological decisions may have suboptimal consequences for both decision makers and the society as a whole. As a particularly emblematic example, consider a hypothetical deontological world in which nobody lies, regardless of consequences, compared to a corresponding consequentialist world in which all people lie if the consequences of lying are good for themselves and for the society as a whole (lies which benefit all parties involved are usually called *Pareto white lies* [3–5]). By definition, both the individual and the society as a whole would be worse off in the deontological world than in the competing consequentialist world. Of course, this argument is not restricted to the case of lying but applies to any moral rule X: a deontological world in which people follow X regardless of consequences would soon be invaded by people who follow X whenever following X benefits all parties involved, and do otherwise if not. In light of these and similar considerations, why do deontological choices persist in human societies?

Several explanations have been put forward. One stems from the remark that consequentialist judgments require the ability to evaluate all possible alternatives in a window of time that is often prohibitive for the limited cognitive capabilities of humans. In the hypothetical consequentialist world introduced above, its inhabitants would have great difficulty to assess whether lying or telling the truth is more advantageous–simply because it is beyond their cognitive capacities to see and evaluate all the consequences of their actions. This implies that the moral value of an action may in practice be inaccessible if one adopts a consequentialist view. This practical inaccessibility makes the development of heuristics, simple short-cuts and rules of thumb, that generally work well on common circumstances, plausible [6]. Following this line of argument, it has been proposed that “deontological philosophy, rather than being grounded in moral *reasoning*, is to a large extent an exercise in moral rationalization” [7]. (See also [8–9]). From this point of view, deontological ethics is not inconsistent with an evolutionary framework, as it may have developed as a set of simple rules that allows us to make reasonably good decisions with little effort, in situations in which it is practically impossible to assess all the consequences of one’s actions [10]. Even more radically, it has been argued that “outside the very narrow domain in which consequences can be unambiguously anticipated, it is not clear at all that calculation processes optimize the outcomes” [11]. See [12] for a criticism of this radical position. In any case, consistent with this general viewpoint, empirical studies have repeatedly shown that intuitive judgments tend to be characteristically deontological, whereas characteristically consequentialist judgments tend to result from deliberative cognitive processes [13–15]; that emotional engagement drive deontological choices in personal dilemmas [16]; that enhanced accessibility of consequentialist outcomes boosts utility maximization [17] and can account for the recent finding that people believe that autonomous vehicles should be utilitarian, while reporting they would buy a (non-utilitarian) car that protects the passenger over other people [18–19].

Another view maintains that deontological rules allow decision makers to avoid moral condemnation and punishment. In a world in which third parties judge, condemn, and punish particular actions, avoiding these actions may be an optimal strategy for avoiding condemnation [20]. But then, why do people condemn and punish certain behaviors, rather than others? It has been proposed that moral judgment is primarily designed as a dynamic coordination device to take sides during conflicts. Specifically, according to this view, each individual comes with a set of moral wrongs, each of which equipped with a magnitude, representing the wrongness of the respective wrong. When a conflict starts, third parties choose sides against the individual who has chosen the action with the greatest wrongness magnitude. Morally motivated punishment serves to signal which side the punisher is on. This mechanism gives rise to a secondary strategic game in which individuals try to influence the set of moral rules to serve their interests. Rawlsian moral rules (i.e., moral rules that people would choose if they did not know their own identity in society, e.g., do not kill) are favored by most people and thus are the most common. However, other moral rules may spread due to the competitive advantages they provide to groups, and they may even change over time within and between cultures due to competition within and between groups. This may explain why the sets of moral wrongs appear not to be universal [21].

However, the main focus of this work is a somewhat different account that has recently originated from the observation that people making deontological judgments may receive indirect evolutionary benefits, as they display (at least) two features that might signal commitment to prosociality [22]. This implies that people making deontological choices may have better chances to be selected as social partners, which, in turn, brings obvious long-term benefits, for example, due to direct and indirect reciprocity [23–24].

The first of these features is that deontological judgments are driven by the explicit prohibition of certain actions, regardless of their consequences. For example, typically deontological rules are: Don’t lie, Don’t steal, Don’t harm–regardless of consequences. The path through which commitment to follow these rules regardless of consequences may favor social interactions is particularly well described by the following example from [22]: an individual who claims that stealing is always wrong will be less likely to steal from me than an individual who believes that stealing may be morally acceptable, depending on its consequences. Thus, people making deontological judgments may be particularly attractive, since potential partners know that they are unlikely to be damaged by them. Direct support for this interpretation comes from recent empirical studies, which uncovered that people making deontological judgments are perceived as being guided less by their self-interest, as being more trustworthy, and as expressing morally stronger views [22, 25–26]. Additional indirect support comes from the work showing that deciding not to tell a Pareto white lie (a characteristically deontological behavior) is positively correlated with altruism and cooperation in economic games [5].

Second, making consequentialist judgments often requires the suppression of strong emotional responses driven by socially desirable values. For example, sacrificing one life to save a greater number of lives requires overriding an emotional response guided by harm aversion–and pushing towards the deontological decision. Being incapable of overriding such emotional responses may favor partner selection along a path similar to the aforementioned one: people displaying strong emotional aversion to harm others will be less likely to harm me, which makes them attractive social partners. Symmetrically, the typically consequentialist emotion of compassion towards all humankind may not be perceived as socially desirable: people helping everyone in the world will be less likely to help me, which makes them little attractive as social partners. In line with this interpretation, recent experimental studies have demonstrated that deontological judgments are positively correlated with harm aversion, and negatively correlated with anti-social personality traits [27–30], and that people making deontological judgments are rated as being more empathic and having a superior moral character [31], as well as warmer [32–33], compared to those who make consequentialist decisions.

In sum, deontological decisions may be favored if they work as a mechanism to signal social desirability.

Of course, this mechanism would be evolutionarily favorable only if people preferring the deontological course of action in a given dilemma are actually more socially desirable than people preferring the competing consequentialist course of action. Otherwise, potential partners would ultimately learn that people making deontological choices in that dilemma are not socially more desirable than those making consequentialist choices, which would eventually lead to the loss of deontologists’ evolutionary advantage. However, although previous research has reported that people making deontological decisions in some dilemmas are *perceived* to be more attractive than people making consequentialist decisions along a number of measures of social desirability, little is known about the direct question: are people making deontological judgments in these dilemmas *actually* more desirable social partners? In particular, to the best of our knowledge, no one has explored this question using the methodology of economic games. (A very recent paper makes use of similar methodology: they compare actual behavior of people making deontological decisions with actual behavior of people making consequentialist decisions, but only in *hypothetical* games [34]. We will discuss their work and its implications in the discussion).

Here we wish to move a first step in this direction.

### Study overview

We investigate whether behavior in moral dilemmas, i.e. having to choose between a characteristically deontological and a characteristically consequentialist option in a fictitious situation, can significantly predict prosocial behavior, which we see as one of the main ingredients of actual social desirability. Prosociality has its manifestation in various forms–with trustworthiness and altruism as some of its primary instantiations. We will test for these using economic games: We examine trustworthiness and altruism with one-shot trust games (henceforth TG) and dictator games (henceforth DG), respectively. We will shortly introduce the TG and the DG, and then move on to a brief discussion of our moral dilemma–a version of the well-known trolley problem–as it was presented to the participants.

#### Trust game (TG)

Two players, Player A and Player B, are paired anonymously. Player A is given $0.20 and has to decide whether or not to transfer it to Player B. If Player A decides to transfer their money to Player B, then Player B receives $0.60. If this happens, Player B is then asked how much of this $0.60, if any, she wants to transfer back to Player A. Then the game ends. (We chose to use small stakes for two reasons. First of all, these stakes are essentially the same as the ones used in [22], which represents the starting point of our analysis. Moreover, previous research has found that DG-altruism [35–36] and TG-trustworthiness [37] are stake-independent, at least as long as stakes are not *too high*–some studies indeed suggest that these prosocial motivations may decrease at very high stakes [36,38]).

It is clear that Player A’s best strategy depends on her beliefs about the amount that Player B is going to return: If Player A believes that Player B is going to return more than $0.20, then she is better off by transferring the money, otherwise, she is better off by keeping the money. For this reason, Player A’s behavior in the Trust Game is considered as an individual measure of trust, and Player B’s behavior is taken as an individual measure of trustworthiness [22,39–40].

#### Dictator game (DG)

Two players, Player A and Player B, are paired anonymously and Player A (the dictator) gets $0.20. Player A can then decide to transfer an amount of their $0.20 to Player B (available options: $0.00, $0.02, $0.04, …, $0.20). Player B has no active role and the money is distributed as proposed by Player A.

Since Player A has no incentive to transfer their money, and since Player B has no possibility to reciprocate Player A’s action, Player A’s donation is usually taken as an individual measure of altruism [41–43].

#### Trapdoor dilemma (TD)

In the TD, participants read the following scenario: “A runaway trolley is heading down the tracks toward five workers who will all be killed if the trolley proceeds on its present course. Adam is on a footbridge over the tracks, in between the approaching trolley and the five workers. Next to him on this footbridge is a stranger who happens to be very large. The only way to save the lives of the five workers is to flip a switch to release a Trapdoor that will drop the stranger off the bridge and onto the tracks below where his large body will stop this trolley. The stranger will die if Adam does this but the five workers will be saved. Participants are asked to report what they think Adam should do in this situation.”

There are theoretical motivations for choosing this dilemma over more classical ones, such as the Trolley problem, or the Footbridge dilemma. On the one hand, the TD allows us to discriminate among people who violate Kant’s practical imperative that humans should never be used solely as a means from those who do not violate this imperative, by, at the same time, avoiding the confounding of emotional salience that is present for example in the footbridge dilemma [22,44]. This is crucial, because, as observed by Everett and colleagues [22], the footbridge dilemma “highlights the possibility that deontologists are simply more averse to physical harm, and not necessarily that they are more reliable cooperators”. On the other hand, the rationale for employing the TD instead of the classical Trolley problem is that the consequentialist choice in the TD requires a more blatant violation of Kant’s imperative than it is the case in the Trolley problem. In line with this intuition, it has been shown that, while deontological judgments in the TD work as a signal of trustworthiness, deontological judgments in the Trolley problem do not [22]. In agreement with these results, in a Pilot Study, we also found that deontological decisions in the Trolley problem do not have a significant effect on neither trustworthiness, nor expectation of other’s trustworthiness (N = 246, all p’s > 0.7).

## Study 1: Trapdoor-deontologists are perceived to be more trustworthy than Trapdoor-consequentialists, but they are actually not

We start by exploring whether people making deontological decisions in the Trapdoor dilemma (Trapdoor-deontologists) are more trustworthy than those making consequentialist decisions in the same dilemma (Trapdoor-consequentialists).

We thus have to test for deontological judgment and for trustworthiness. Concerning the former, we present subjects with the Trapdoor dilemma. Concerning the latter, we measure a person’s perception of others’ trustworthiness by having her play a TG in the role of Player A, and we test a person’s trustworthiness by having her play a TG in the role of Player B.

The aim of our first study is to replicate the finding that Trapdoor-deontologists are perceived to be more trustworthy than Trapdoor-consequentialists [22,34] *and* to explore whether Trapdoor-consequentialists are actually less trustworthy than Trapdoor-deontologists.

### Hypotheses

*H1*.*1*. Trapdoor-deontologists are perceived to be more trustworthy than Trapdoor consequentialists.

*H1*.*2*. Trapdoor-deontologists are actually more trustworthy than Trapdoor-deontologists.

### Method

#### Participants

We recruited 300 American participants using Amazon Mechanical Turk (AMT) [45–48], to participate on an incentivized online survey that we prepared using Qualtrics. The experiments reported in this paper were conducted in 2016, when all authors were based at the University of Amsterdam. According to the Dutch legislation, this is a non-WMO study, that is (i) it does not involve medical research and (ii) participants are not asked to follow rules of behavior. See http://www.ccmo.nl/attachments/files/wmo-engelse-vertaling-29-7-2013-afkomstig-van-vws.pdf, §1, Article 1b, for an English translation of the Medical Research Act. Thus (see http://www.ccmo.nl/en/non-wmo-research) the only legislations which apply are the Agreement on Medical Treatment Act, from the Dutch Civil Code (Book 7, title 7, §5), and the Personal Data Protection Act (a link to which can be found in the previous webpage). The current study conforms to both. Implied consent via survey was obtained by all subjects prior to participating and data were fully anonymized before analysis. The participation fee was $0.50. Participant could also earn additional money depending on the choice they make during the experiment, as detailed in the *Design* subsection. After the survey was completed, we downloaded the datafile from Qualtrics, which contained 370 observations. The number of observations resulting on Qualtrics is usually higher than the number of submissions on AMT as Qualtrics includes observations of subjects that get excluded along the survey because they fail to correctly answer the comprehension questions. A total of 130 subjects were excluded because they either failed the comprehension questions or took the survey more than once, leaving us with a final sample of 240 participants and valid surveys. The number of participants who pass the comprehension questions is never equal to the number of actual submissions on AMT, because some subjects submit their survey on AMT even if they are eliminated along the survey. The proportion of participants who were excluded is in line with previous experiments using economic games on AMT [46]. For this and the subsequent studies we did not conduct an a priori power analysis, but sample sizes were based on earlier studies testing behavioral changes in economic games involving prosociality. In Study 1, we recruited 100 participants per condition. In the subsequent studies, to increase power, we recruited 150 participants per condition. Data of each study were collected all together and analyzed after the experiment. Data collection was not continued after data analysis. All measures, manipulations, and exclusions in this and the following studies are disclosed.

#### Design

Participants were randomly assigned to one of three conditions. In the *PlayerB-Trapdoor-Consequentialist* condition, participants played a Trust Game in the role of Player A with a Trapdoor-consequentialist in the role of Player B. More precisely, participants were shown the instructions of the Trapdoor dilemma and were informed that a participant who had already completed the survey, named Player B, opted for the consequentialist course of action. A comprehension question (regarding what happens if Adam flips the switch) was asked to make this point as clear as possible. Participants failing this comprehension question were automatically excluded from the survey. Participants in this condition were not asked to make a choice in the Trapdoor dilemma. They were only informed about Player B’s action. Subsequently, participants were shown the instructions of the Trust game. After reading these instructions and before making a choice, participants were asked four comprehension questions (regarding the actions that maximize players’ payoffs in four different scenarios). Participants failing any comprehension question were automatically excluded from the survey. The *PlayerB-Trapdoor-Deontologist* condition was similar to the previous condition, but participants played a Trust Game in the role of Player A with a Trapdoor-deontologist in the role of Player B. In the *Trapdoor-PlayerA* condition, participants first made a choice in the Trapdoor dilemma, and then played a Trust Game in the role of Player B with one Player A who had decided to transfer their $0.20. After the survey was completed, we matched participants according to their choices and according to the conditions they participated in, we computed the bonuses and we paid them on top of their participation fee.

#### Results and discussion

**Fig 1:**
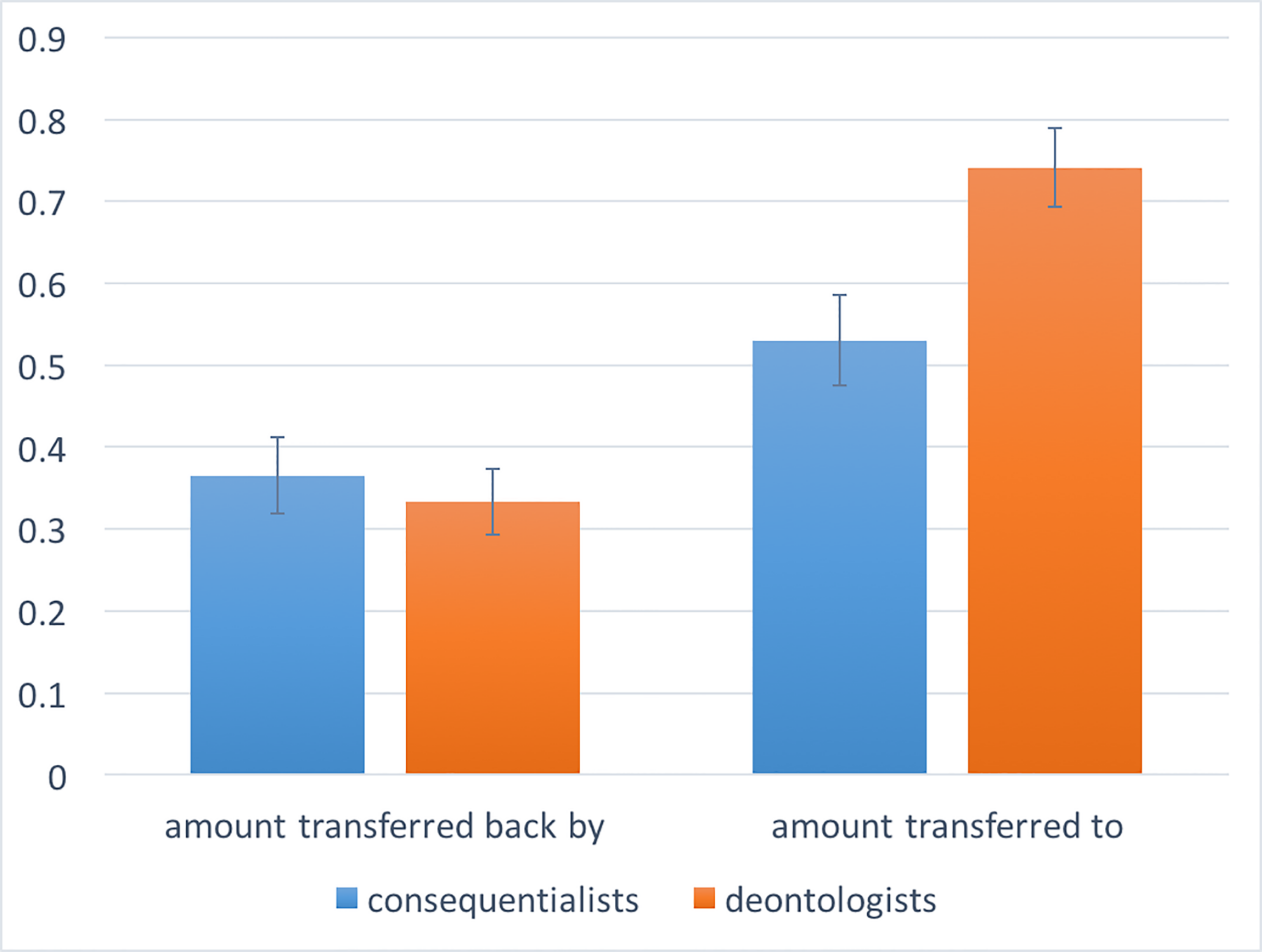
Deontologists are perceived to be more trustworthy than consequentialists, but they are actually not. The pair of columns on the left-hand side reports the average amount transferred back by Player B to Player A in the Trust Game as a function of whether Player B is a Trapdoor-deontologist or a Trapdoor-consequentialist. The pair of columns on the right-hand side reports the average amount transferred by Player A to Player B, as a function of whether Player B is a Trapdoor-deontologist or a Trapdoor-consequentialist. Error bars represent the standard error of the mean.

The N = 240 subjects who passed the comprehension questions were distributed across conditions as follows: N = 83 in the *PlayerB-Trapdoor-Consequentialist* condition; N = 85 in the *PlayerB-Trapdoor-Deontologist* condition; and N = 72 in the *Trapdoor-PlayerA* (34 of whom chose the consequentialist option, while the remaining 38 chose the deontological option). Fig 1 provides visual evidence that Trapdoor-deontologists were perceived to be more trustworthy than Trapdoor-consequentialists, but they were actually not. To show this, we define a variable “prosocial” which: for those who participated as Player A, it takes value 1 if they transferred their $0.20 to Player B, and 0 otherwise; for those who participated in the role of Player B, it measures the amount returned to Player A, normalized such that the maximum return, which is $0.60, corresponds to 1. Linear regression predicting Prosocial as a function of three dummy variables, Player A (1 if a subject participated as Player A, and 0 otherwise), Consequentialist (1 if a subject was/was-paired-with a Trapdoor-consequentialist, and 0 otherwise), and their interaction, reveals indeed a significant interaction (F(3,236) = 11.50, coeff = -0.24, t = -2.05, p = 0.041). We now look at main effects. We find that the participants playing the TG in the role of Player A transferred significantly more to Trapdoor-deontologists than to Trapdoor-consequentialists playing as Player B (69.2% vs 57.7%, F(1,166) = 8.40, coeff = 0.21, t = 2.90 p = 0.004). This suggests that Trapdoor-deontologists were expected to be more trustworthy than Trapdoor-consequentialists. However, the amount returned by Trapdoor-deontologists is not significantly different than the amount returned by Trapdoor-consequentialists (33.2% vs 34.4%, F(1,70) = 0.27, coeff = -0.03, t = -0.52, p = 0.602). This suggests that Trapdoor-deontologists were not actually more trustworthy than Trapdoor-consequentialists. We also conducted a Bayesian hypothesis test [49–50] as follows: we conducted linear regression predicting Pro-sociality with and without the Consequentialist variable to compute the corresponding BICs and we used these values to compute the posterior probability of the null hypothesis (no differences between trapdoor-consequentialists and trapdoor-deontologists) given the prior that the null hypothesis and the alternative hypothesis are equally likely. In doing so, we found a posterior probability of 92.23%, which provides indeed strong support for the null hypothesis that there is no difference in trustworthiness between Trapdoor-consequentialists and Trapdoor-deontologists.

#### Conclusion

Trapdoor-deontologists are perceived to be more trustworthy in the Trust Game than Trapdoor-consequentialists, but actually they are not.

## Study 2: Trapdoor-deontologists are perceived to be more altruist than Trapdoor-consequentialists, but they are actually not

Study 1 uncovered a perception gap according to which Trapdoor-deontologists are not significantly more trustworthy than Trapdoor-consequentialists, although they are perceived to be so.

However, trustworthiness is only one particular dimension of prosociality. Study 2 aims at exploring what happens if we adopt altruistic behavior instead of trustworthiness as a measure of prosociality. (As in Study 1, also in this case we conducted a Pilot Study with the standard Trolley problem, instead of the Trapdoor dilemma. And, also in this case, we found no statistically significant effect: Trolley-deontologists were neither perceived to be significantly more altruistic than Trolley-consequentialists, nor they were significantly more altruistic than Trolley-consequentialists).

### Hypotheses

*H2*.*1*. Trapdoor-deontologists are perceived to be more altruistic than Trapdoor-consequentialists.

*H2*.*2*. Trapdoor-deontologists are actually more altruistic than Trapdoor-consequentialists.

### Method

#### Participants

1,050 American participants (none of which had participated in the previous study) were recruited using AMT. They were paid $0.50 as a participation fee and they could earn additional money depending on the choice they make during the experiment, as detailed below. After the survey was completed, we downloaded the datafile from Qualtrics, which contained 1,079 observations. A total of 238 subjects were excluded because they either failed the comprehension questions or took the survey more than once, leaving us with a final sample of 841 participants.

#### Design

Participants were randomly divided between three conditions. In the *Guess-Trapdoor-Consequentialist* condition, participants had to guess the DG donation of a randomly selected Trapdoor-consequentialist participant, with a $0.20 reward in case they make the right guess. More precisely, participants were shown the Trapdoor dilemma and were informed that another participant, named Player A, who had already completed the survey, had chosen the consequentialist option. One comprehension question was asked to make this point as clear as possible. Participants failing this comprehension question were automatically excluded from the survey. Participants in this condition were not asked to make a decision in the Trapdoor dilemma. They were only informed about Player A’s choice. Subsequently, participants were informed that Player A was playing a DG with a third participant, named Player B. After reading the instructions of the DG, participants were asked two comprehension questions (one regarding which action maximizes the dictator’s payoff, and the other one regarding which action maximizes the recipient’s payoff). Participants failing any comprehension question were automatically excluded from the survey. Participants who passed the comprehension questions were asked to guess Player A’s donation to Player B. The *Guess-Trapdoor-Deontologist* condition was similar to the previous condition, with the difference that participants had to guess the DG donation of a Trapdoor-deontologist participant. In the *DG-Trapdoor* condition, participants first made a choice in the Trapdoor Dilemma and then in the DG. (In reality, participants were divided in seven conditions, because each “Guess condition” was actually made by three conditions: the one described, and two more conditions in which participants were informed about the gender of the donor. However, knowing the gender did not have any significant impact on guesses, and thus we collapse across conditions). After the survey was completed, we paired participants according to their decisions and we computed and paid their bonuses.

#### Results and discussion

**Fig 2:**
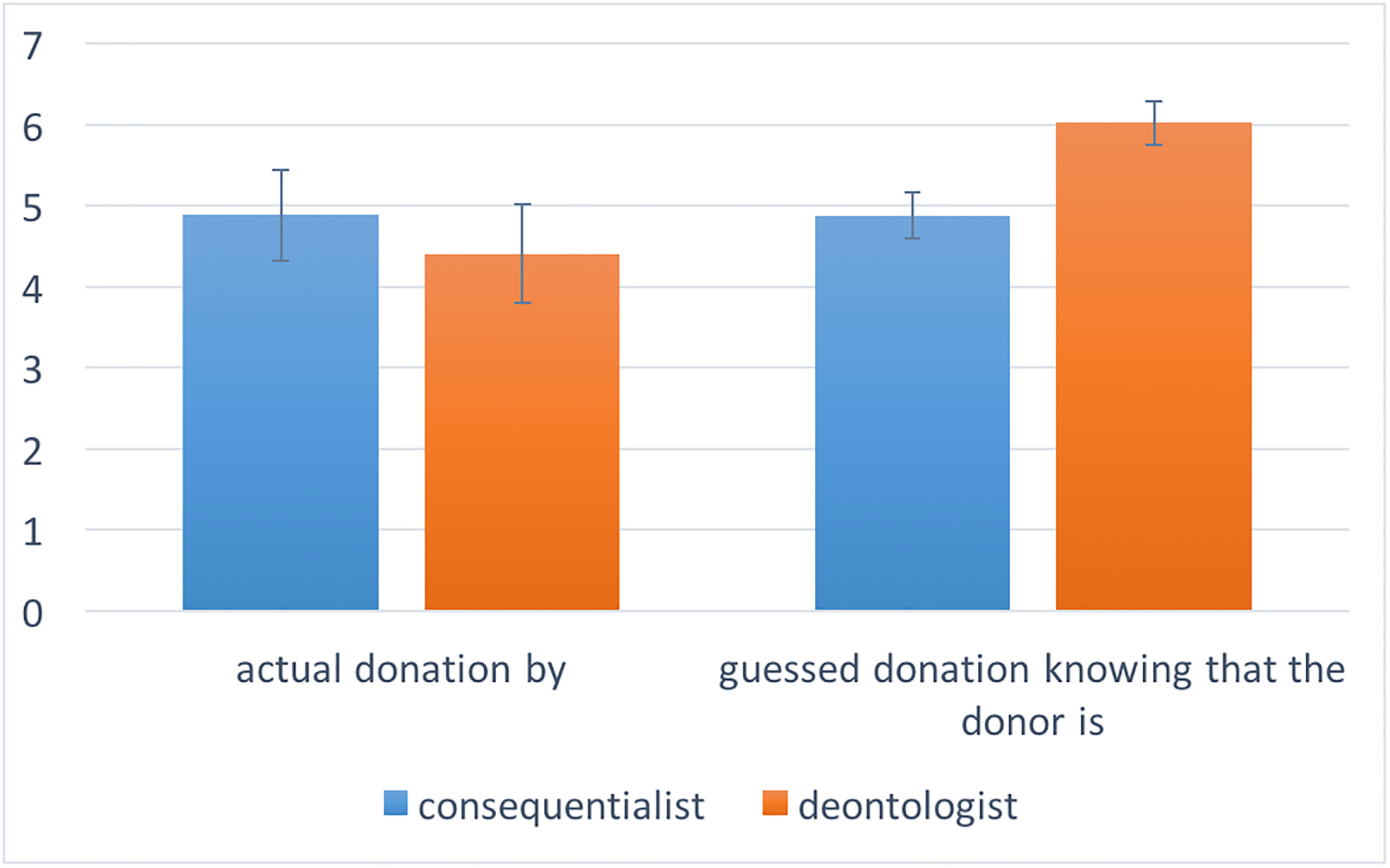
Deontologists are perceived to be more altruistic than consequentialists, but they are actually not. The pair of columns on the left-hand side represents the average donation made by dictators as a function of whether they are Trapdoor-consequentialists or Trapdoor-deontologists. The pair of columns on the right-hand side represents the average donation guessed by observers as a function of whether the donor is a Trapdoor-consequentialist or a Trapdoor-deontologist. Error bars represent the standard error of the mean.

The N = 841 subjects were distributed across conditions as follows: N = 326 in the *PlayerB-Trapdoor-Consequentialist* condition; N = 369 in the *PlayerB-Trapdoor-Deontologist* condition; and N = 146 in the *Trapdoor-PlayerA* (69 of whom choose the consequentialist option, while the remaining 77 choose the deontological option). Fig 2 provides visual evidence that Trapdoor-deontologists were perceived to be more altruistic than Trapdoor-consequentialists, but actually they were not more altruistic. In line with Study 1, linear regression predicting prosocial behavior as a function of Player A, Consequentialist, and their interaction, reveals a marginally significant interaction (F(3,837) = 4.08, coeff = -.08, t = -1.74, p = 0.081). Looking at main effects, in line with Study 1, we find that the participants expected Trapdoor-deontologists to give significantly more than Trapdoor-consequentialists (30% of the total share vs 24%, F(1,166) = 8.40, coeff = -.21, t = -2.90, p = 0.004). However, the actual donation of Trapdoor-deontologists was not significantly different than the actual donation of Trapdoor-consequentialists (22% vs 24%; F(1,144) = 0.34, coeff = .024, t = 0.58, p = 0.563). We also conducted a Bayesian hypothesis following a similar procedure as in Study 1. In doing so, we found a posterior probability of 91.06%, which provides indeed strong support for the null hypothesis that there is no difference in altruistic behavior between Trapdoor-consequentialists and Trapdoor-deontologists.

#### Conclusion

Trapdoor-deontologists are perceived to be more altruistic in the Dictator Game than Trapdoor-consequentialists, but they are not actually so.

## General discussion

We tested the hypothesis that people making deontological judgments in the Trapdoor dilemma are more trustworthy and more altruistic towards strangers than those making consequentialist judgments. In doing so, we found a perception gap such that people perceive Trapdoor-deontologists to be more altruistic and more trustworthy towards strangers than Trapdoor-consequentialists, but they actually are not.

Our results are naturally situated in a framework that stems from evolutionary considerations. As mentioned above, deontological judgments often lead to positive character evaluations which, in turn, can lead to evolutionary advantages when it comes to partner choice mechanisms [22,51–54]. Our findings indicate that this procedure might not promote favorable outcomes as the positive character evaluation itself is not manifested in prosocial behavior–at least when prosociality is operationalized using trustworthiness in the TG and altruism in the DG. It is thus reasonable to wonder: how and why did people’s perception concerning the selection of their social partners evolve in order to favor deontological decisions?

This point highlights the main limitation of our study. We operationalized social desirability using prosociality in the trust game and the dictator game. (We have also conducted a pilot using the Prisoner’s Dilemma (PD), as a measure of cooperative behavior towards strangers [55–57]. Again we found that Trapdoor-consequentialists are neither significantly more nor significantly less cooperative than Trapdoor-deontologists. However, in this pilot, we did not measure beliefs. So, we do not know whether the perception gap discussed in Study 1 and Study 2 extends to cooperative behavior in PD). This procedure is inherently limited, as it does not take into account a myriad of other, potentially different, behaviors of crucial importance in social relationships. Although previous research has shown that TG-trustworthiness and DG-altruism correlate with a number of other prosocial behaviors in the lab [40, 58–60], and in real-life [61–62], it is possible that using other measures of prosociality would lead to different results. For example, one may wonder whether deontological judgments in the Trapdoor dilemma, while not predicting prosociality towards anonymous strangers, work as a signal of commitment to prosociality towards people belonging to the same social group–the so-called *ingroup favoritism* [63–64]. This is a promising stream of research because consequentialism is radically impartial, while deontological ethics focus on notions of duties, rights, and obligations, which may be context-dependent. Thus, everything else being constant, Trapdoor-deontologists might favor ingroup members over outgroup members to a larger extent than consequentialists do. In line with this, a recent work found that people prefer those making deontological choices (in a dilemma between volunteering for cause or helping a family member) as a friend or spouse, but prefer those making consequentialist choices as political leaders [22]. In any case, an important direction for future research is to expand our study to include other economic games and thereby examining in more depth how expectations of prosocial actions of Trapdoor-deontologists and their actual behaviors are connected in different contexts.

Additionally, we operationalized social desirability using *actual* behavior, and this led us to use economic games. This procedure has two limitations. First, actual behavior in economic games pertains to the domain of prescriptions, while judgments in moral dilemmas pertain to the domain of proscriptions [65–66]. Since it is possible that the psychology underlying decisions is different from the psychology underlying judgments [30], this might be at the origin of the observed gap between expectations and actual behavior. Second, it is possible that Trapdoor-deontologists are more desirable than Trapdoor-consequentialists along dimensions that are not easily measurable using economic games, as, for example, warmth and empathy, as some recent studies using different moral dilemmas seem to suggest [32–33]. Thus, another interesting strand of further research is to extend our study to include other scales of social desirability, centered around proscriptions, instead of prescriptions, also including psychometric measures [67–68]. Symmetrically, we measured deontological judgment using a hypothetical Trapdoor dilemma. However, previous research suggests that there might be significant disparities between judgment and actual behavior in moral dilemmas [69–70]. This ultimately suggests that it is possible that deontological choices become an honest signal of prosociality if less hypothetical measures of deontology are used. Exploring this possibility is an important direction for future research.

A similar point regards our measure of deontological judgment. As in [22], we operationalized deontological judgment by using the Trapdoor dilemma, because this dilemma allows us to discriminate among people who violate Kant’s practical imperative from those who do not violate this imperative, by, at the same time, avoiding the confounding of emotional salience that is present for example in the footbridge dilemma. This distinction is crucial, because it has been proposed that deontological judgments signal commitment to prosociality through signaling commitment to follow Kant’s practical imperative that other people should never be used solely as a means [16]. The choice of the Trapdoor dilemma has, however, also two limitations. One is that this and similar dilemmas have been criticized for evoking humor, rather than serious consideration of moral concerns [71]. The second one is that the practical imperative is not the only dimension in which deontological ethics differs from consequentialism. Thus, in future research, it would be important to extend our study to include more dimensions of deontological judgment, in order to determine the boundary conditions of our results. We have done a first step in this direction. As mentioned earlier, in line with [22], in a Pilot Study, we have also found that deontological judgments in the standard Trolley problem do *not* signal trustworthiness. Understanding which deontological judgments signal pro-sociality and which give rise to the observed gap between expectations and behavior is certainly an important direction for future research, that may shed light on which moral principles are involved in prosocial behavior and in the perception of others’ prosocial attitudes.

Another potential source of criticism is the use of small stakes. We chose to use such small stakes for two reasons. First of all, we used essentially the same stakes as in [22], which represents the starting point of our analysis. Second, previous research has found no stake effect on DG-altruism [35–36] and TG-trustworthiness [37]–some studies suggest that these prosocial motivations may decrease at very high stakes [35,38]. Thus we believe that it is unlikely that future research will reveal that Trapdoor-consequentialists becomes less trustworthy and altruistic than Trapdoor-deontologists, at least when using standard medium-size stakes. Potentially more intriguing is what could happen at very large stakes, as previous research suggests that prosocial motivations may decrease in such situations. It is possible that this decrease in prosocial motivations is driven by consequentialists and that, at very large stakes, Trapdoor-consequentialists become indeed less trustworthy and altruistic than Trapdoor-deontologists.

Nonetheless, we should say that we *did* find a significant gap between expectations and actual behavior. Even though future research may uncover that people making deontological decisions are actually more prosocial than those making consequentialist decisions along other dimensions of prosociality or at higher stakes, our study shows that, as a matter of fact, in our tasks, Trapdoor-deontologists are perceived to be more trustworthy and more altruistic than Trapdoor-consequentialists, although they are actually equally trustworthy and equally altruistic. Our results are, however, silent regarding the psychological underpinnings behind this gap. We hope that future research may shed light on *why* Trapdoor-deontologists are perceived to be more prosocial than Trapdoor-consequentialists, in spite of not being so.

Therefore, besides the aforementioned limitations, our work has also several positive implications. For example, the result that respecting Kant’s practical imperative in the Trapdoor dilemma is perceived as a signal of altruism and trustworthiness is theoretically remarkable as it suggests that people believe that Kant’s practical imperative is one of the determinants of prosociality, and thus that prosociality and morality are linked, at least in some contexts. This mirrors recent findings that morality drives prosociality in a variety of contexts [72–74], and suggests that exploring the links between morality and prosociality can be a fruitful avenue for future research. Designing the boundaries of our results along the dimensions of the deontology and prosociality measures are not *just-academic* questions, but can elucidate underlying theoretical relations between motivations, behaviors, and beliefs that have been left uncovered by previous research. These links can turn out to be useful to find novel descriptions of people’s behavior and people’ expectations in other people’s behavior in terms of underlying moral principles.

A very recent work is the most similar to ours that we are aware of [34]. They compared the actual behavior of people making deontological judgments and people making consequentialist judgments in a hypothetical Trust Game and in a hypothetical 4-player Public Goods Game (PGG, which represents a way to measure cooperative behavior in groups of four people). In line with our study, [34] found that people making deontological judgments are perceived to be more trustworthy than people making consequentialist judgments while, actually, they are not. However, they found that people making deontological judgments are perceived to be more cooperative in the hypothetical 4-player Public Goods game than people making consequentialist judgments *and* actually are more cooperative–there is thus no perception gap observable in the PGG. Thus, our work extends the result of [34] along two dimensions: first, we show that Trapdoor-deontologists are perceived to be more trustworthy than Trapdoor-consequentialists in a real, incentivized Trust Game, rather than a hypothetical one. This is an important extension, because economic motivations can be an important drive of human behavior, especially among people making consequentialist choices. Second, we showed that this perception gap between the expected level of prosociality of Trapdoor-deontologists versus Trapdoor-consequentialists and their actual levels of prosociality does not only regard the domain trustworthiness, but also that of altruistic behavior towards strangers.

A somewhat related work is [75]. Here the authors asked subjects to self-report the motivation behind their choice in a dictator game, and then classified the responses in several classes, including to whether they were consequentialist or deontologist. The authors found no differences in dictator game giving between subjects who left a consequentialist motivation and those who left a deontological motivation. This result is thus in line with the current results of ours. Our work extends [75] along two directions: first, we do not only consider altruistic behavior in the dictator game, but we also consider trustworthiness in the trust game; second, we do not use self-report motivations to classify subjects into consequentialists or deontologists, but we use actual judgments in a specific moral dilemma, that we have chosen because of specific theoretical motivations.

In sum, deontological judgments do not work as a universal reliable signal of prosociality. Understanding if, when, and why deontological judgments correlate with prosocial behavior is an important direction for future research with important consequences for our understanding of human sociality.
