# Correction: People making deontological judgments in the Trapdoor dilemma are perceived to be more prosocial in economic games than they actually are

**DOI:** 10.1371/journal.pone.0225850

**Published:** 2019-11-21

**Authors:** Valerio Capraro, Jonathan Sippel, Bonan Zhao, Levin Hornischer, Morgan Savary, Zoi Terzopoulou, Pierre Faucher, Simone F. Griffioen

In Fig 1, the columns that depict the average amount transferred by Player A to Player B as a function of whether Player B is a Trapdoor-deontologist or a Trapdoor-consequentialist report incorrect values. The average amounts should be 57.7% and 69.2%. Please see the correct Fig 1 here.

**Fig 1:**
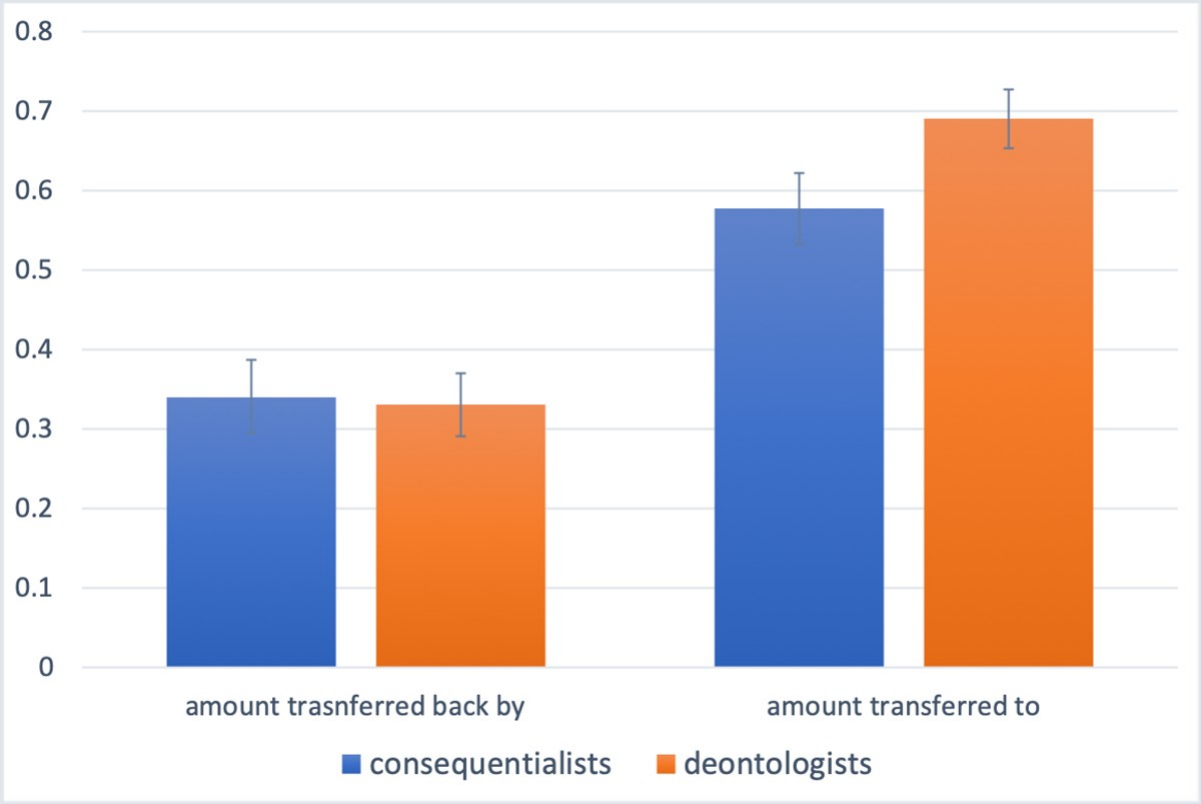
Deontologists are perceived to be more trustworthy than consequentialists, but they are actually not. The pair of columns on the left-hand side reports the average amount transferred back by Player B to Player A in the Trust Game as a function of whether Player B is a Trapdoor-deontologist or a Trapdoor-consequentialist. The pair of columns on the right-hand side reports the average amount transferred by Player A to Player B, as a function of whether Player B is a Trapdoor-deontologist or a Trapdoor-consequentialist. Error bars represent the standard error of the mean.
